# Influence of the HDAC Inhibitor Valproic Acid on the Growth and Proliferation of Temsirolimus-Resistant Prostate Cancer Cells In Vitro

**DOI:** 10.3390/cancers11040566

**Published:** 2019-04-19

**Authors:** Jasmina Makarević, Jochen Rutz, Eva Juengel, Sebastian Maxeiner, Igor Tsaur, Felix K.-H. Chun, Jürgen Bereiter-Hahn, Roman A. Blaheta

**Affiliations:** 1 Department of Urology, Goethe-University, D-60590 Frankfurt am Main, Germany; jmakarevic@air-net.de (J.M.); Jochen.Rutz@kgu.de (J.R.); Eva.Juengel@unimedizin-mainz.de (E.J.); SebastianMaxeiner@gmx.de (S.M.); igor.tsaur@unimedizin-mainz.de (I.T.); Felix.Chun@kgu.de (F.K.-H.C.); 2 Institute for Cell Biology and Neurosciences, Goethe-University, D-60590 Frankfurt am Main, Germany; Bereiter-Hahn@bio.uni-frankfurt.de

**Keywords:** mtor, HDAC, cell growth, cdk, cyclins, prostate cancer, resistance, valproic acid, temsirolimus

## Abstract

The mechanistic target of rapamycin (mTOR) is elevated in prostate cancer, making this protein attractive for tumor treatment. Unfortunately, resistance towards mTOR inhibitors develops and the tumor becomes reactivated. We determined whether epigenetic modulation by the histone deacetylase (HDAC) inhibitor, valproic acid (VPA), may counteract non-responsiveness to the mTOR inhibitor, temsirolimus, in prostate cancer (PCa) cells. Prostate cancer cells, sensitive (parental) and resistant to temsirolimus, were exposed to VPA, and tumor cell growth behavior compared. Temsirolimus resistance enhanced the number of tumor cells in the G2/M-phase, correlating with elevated cell proliferation and clonal growth. The cell cycling proteins cdk1 and cyclin B, along with Akt-mTOR signaling increased, whereas p19, p21 and p27 decreased, compared to the parental cells. VPA significantly reduced cell growth and up-regulated the acetylated histones H3 and H4. Cdk1 and cyclin B decreased, as did phosphorylated mTOR and the mTOR sub-complex Raptor. The mTOR sub-member Rictor and phosphorylated Akt increased under VPA. Knockdown of cdk1, cyclin B, or Raptor led to significant cell growth reduction. HDAC inhibition through VPA counteracts temsirolimus resistance, probably by down-regulating cdk1, cyclin B and Raptor. Enhanced Rictor and Akt, however, may represent an undesired feedback loop, which should be considered when designing future therapeutic regimens.

## 1. Introduction

Prostate cancer (PCa) is the second most diagnosed cancer in men and the fifth most frequent cause of death among them worldwide [1]. Treatment for localized prostate cancer with no identifiable regional lymph nodes or distant metastases includes watchful waiting, active surveillance, surgery and radiation. Once metastasized, androgen deprivation therapy has long been considered standard care, either as a monotherapy or, later, combined with taxane-based regimens.

Yet, after 12–18 months of hormonal therapy, resistance inevitably develops in almost all patients [2], leading to castration-resistant prostate cancer (CRPC). To overcome this obstacle, new-generation antiandrogens, such as abiraterone acetate and enzalutamide, have been approved for the treatment of metastatic CRPC [3]. Although new-generation antiandrogens provide a significant benefit for CRPC patients, randomized studies have demonstrated only moderate improvement in median overall survival outcome over placebo, that is, 15.8 vs. 11.2 months for abiraterone, respectively, and 18.4 vs. 13.6 months for enzalutamide, respectively [4].

Therefore, new therapeutic approaches are urgently needed. Developing targeted compounds, more potent than the drug panel actually in use, is one option. Evidence has been provided that the androgen receptor signaling pathway cross-communicates with the phosphatidylinositol 3-kinase/protein kinase B/mechanistic target of rapamycin (PI3K/Akt/mTOR) pathway. In fact, activation of the PI3K/Akt/mTOR signaling pathway occurs in nearly 50% of CRPC cases, making mTOR an attractive therapeutic target [5,6].

Clinical studies have demonstrated a significant benefit of the mTOR inhibitors temsirolimus (Torisel^®^; Wyeth Pharmaceuticals Inc., Philadelphia, PA, USA) and everolimus (Afinitor^®^; Novartis Pharmaceuticals Corporation, East Hanover, NJ, USA) in treating renal cell carcinoma (RCC) patients. It was, therefore, thought that these drugs may also be of value for other solid tumor types. Disappointingly, mTOR blockade in patients with metastatic CRPC has proved only mildly effective [7,8] due to resistance development. A further drug combined with mTOR pathway inhibitors is therefore necessary to combat resistance. This drug must induce longer-lasting anti-tumor effects by profoundly blocking mTOR signaling and simultaneously preventing undesired feedback mechanisms. Based on an RCC cell model, resistance to mTOR inhibition has been counteracted by blocking histone deacetylase (HDAC) activity, pointing to an epigenetic control of Akt-mTOR [9,10]. Indeed, it has recently been documented that the Akt-mTOR axis communicates with HDAC-driven acetylation of histones H3 and H4 in prostate cancer cells [11]. Consequently, combining an mTOR inhibitor with an HDAC inhibitor may optimize an anti-tumor protocol. A dose escalation study on patients with advanced malignancy, treated with the mTOR inhibitor sirolimus and the HDAC inhibitor vorinostat, has revealed preliminary anticancer activity [12]. HDAC inhibition has also been shown to induce a strong response of temsirolimus-resistant bladder and RCC cancer cells [13,14]. Resistance to temsirolimus is associated with increased adhesion and migration of prostate cancer cells, which could be reverted by down-regulating HDAC [15].

The current study was designed to evaluate the potential of valproic acid (VPA) as a prototype HDAC inhibitor of growth and proliferation in temsirolimus-resistant prostate cancer cells by exploring its underlying molecular mode of action.

## 2. Materials and Methods

### 2.1. Cell Culture

The human prostate tumor cell lines PC3, DU-145 (both castration-resistant), and LNCaP (castration-sensitive) were obtained from DSMZ (Braunschweig, Germany). Tumor cells were grown and subcultured in RPMI 1640 medium (Gibco/Invitrogen, Karlsruhe, Germany) supplemented with 10% fetal calf serum (FCS), 2% HEPES (2-[4-(2-hydroxyethyl)piperazin-1-yl]ethanesulfonic acid) buffer (1 M, pH 7.4), 2% glutamine, 1% penicillin/streptomycin at 37 °C in a humidified, 5% CO_2_ incubator. The temsirolimus-resistant sublines developed over 12 months by continuous exposure to temsirolimus (LC Laboratories, Woburn, MA, USA), starting at 1 nmol/mL and increasing stepwise to 1 µmol/mL (PC3^res^, DU-145^res^, LnCaP^res^). The control cells remained untreated (PC3^par^, DU-145^par^, LNCaP^par^).

### 2.2. Drugs

Temsirolimus was dissolved in dimethylsulfoxide (DMSO) as 10 mM stock solution and stored as aliquots at −20 °C. To induce resistance, temsirolimus was diluted in cell culture medium to a final concentration of 1 nmol/mL–10 µmol/mL. Control cell cultures received cell culture medium alone. VPA (G. L. Pharma GmbH, Lannach, Austria) was used at a final concentration of 1 mM. The temsirolimus-resistant and temsirolimus-sensitive cell lines were pre-treated for three days with VPA before growth and proliferation experiments were performed. The response to therapeutic temsirolimus concentrations (drug re-treatment) was also investigated. Preparation for temsirolimus re-treatment was carried out by incubating the resistant tumor cells for three days with temsirolimus-free medium. Subsequently, 10 nmol/mL of temsirolimus were applied to the drug-resistant and drug-sensitive cells, and cell cultures were then subjected to the assays indicated below. To exclude toxic effects of the compounds, cell viability was determined by trypan blue (Gibco/Invitrogen, Karlsruhe, Germany).

### 2.3. Tumor Cell Growth and Proliferation

Cell growth was assessed using the 3-(4,5-dimethylthiazol-2-yl)-2,5-diphenyltetrazolium bromide (MTT) dye reduction assay (Roche Diagnostics, Penzberg, Germany). Drug-resistant and drug-sensitive cells (100 µL, 1 × 10^4^ cells/mL) were seeded onto 96-well tissue culture plates. After 24 h, 48 h and 72 h, MTT (0.5 mg/mL) was added for an additional 4 h. Thereafter, cells were lysed in a buffer containing 10% SDS in 0.01 M HCl. The plates were incubated overnight at 37 °C, 5% CO_2_. Absorbance at 550 nm was determined for each well using a microplate ELISA reader (Tecan Group Ltd., Männedorf, Switzerland). To allow an accurate quantification of the cell number, the relationship between cell number and signal produced was established by a calibration curve. Each experiment was done in triplicate. After subtracting background absorbance, results were expressed as mean cell number.

Cell proliferation was measured using a BrdU cell proliferation enzyme-linked immunosorbent assay (ELISA) kit (Calbiochem/Merck Biosciences, Darmstadt, Germany). PC3^par^ or PC3^res^ cells, seeded onto 96-well microtiter plates, were incubated with 20 µL BrdU-labeling solution per well for 8 h, fixed and detected using anti-BrdU mAb according to the manufacturer’s instructions. Absorbance (optical density, OD) was measured at 450 nm.

To evaluate whether tumor cell growth was impaired or reduced due to apoptosis, the expression of Annexin V/propidium iodide was evaluated using the Annexin V-FITC Apoptosis Detection kit (BD Pharmingen, Heidelberg, Germany). Tumor cells were washed twice with PBS, and then incubated with 5 μL of Annexin V-FITC and 5 μL of propidium iodide in the dark for 15 min at room temperature. Cells were analyzed by flow cytometry using a FACScalibur flow cytometer (BD Biosciences, Heidelberg, Germany). The percentage of apoptotic (early and late), necrotic, and vital cells in each quadrant was calculated using CellQuest software (version 6.0; BD Biosciences).

### 2.4. Clonogenic Growth Assay

Resistant and sensitive tumor cells were transferred to 6-well plates at 500 cells per well. Following 10 days incubation, cell colonies were fixed and counted. Clones of at least 50 cells were counted as one colony.

### 2.5. Cell Cycle Analysis

Cell cycle analysis was carried out using temsirolimus-resistant and temsirolimus-sensitive cultures grown to subconfluency. Tumor cell populations were stained with propidium iodide, using a Cycle TEST PLUS DNA Reagent Kit (BD Biosciences) and then subjected to flow cytometry with a FACScalibur flow cytometer (BD Biosciences). Then, 10,000 events were collected from each sample. Data acquisition was carried out using CellQuest software and cell cycle distribution was calculated using the ModFit software (version 3.0; BD Biosciences). The number of gated cells in G0/G1-, G2/M-, or S-phase was presented as %.

### 2.6. Western Blot Analysis

Cell cycle-regulating proteins were investigated in PC3^par^ and PC3^res^ cells. Tumor cell lysates were applied to a 7% polyacrylamide gel and electrophoresed for 90 min at 100 V. The protein was then transferred to nitrocellulose membranes (1 h, 100 V). After blocking with non-fat dry milk for 1 h, the membranes were incubated overnight with the following monoclonal antibodies: Anti cdk1 (IgG1, clone 1), Anti cdk2 (IgG2a, clone 55), Anti cdk4 (IgG1, clone 97), Anti cyclin A (IgG1, clone 25), Anti cyclin B (IgG1, clone 18), Anti cyclin D3 (IgG2b, clone 1), Anti cyclin E (IgG1, clone HE12) Anti p19 (IgG1, clone 52/p19), Anti p21 (IgG1, clone 2G12) and Anti p27 (IgG1, clone 57) (all were obtained from BD Biosciences). Horseradish peroxidase (HRP)-conjugated goat-anti-mouse IgG (Upstate Biotechnology, Lake Placid, NY, USA; dilution 1:5000) served as the secondary antibody. Membranes were briefly incubated with enhanced chemiluminescence (ECL) detection reagent (ECLTM, Amersham/GE Healthcare, München, Germany) to visualize the proteins and then analyzed by the Fusion FX7 system (Peqlab, Erlangen, Germany). β-actin (1:1000; Sigma-Aldrich, Taufenkirchen, Germany) served as the internal control.

To explore target specificity of temsirolimus and VPA, mTOR signaling and histone acetylation were also evaluated after a 24 h incubation. The following monoclonal antibodies were used:

Anti mTOR (IgG, clone 7C10), Anti phospho mTOR (pmTOR; IgG, Ser2448, clone D9C2), Anti Rictor (IgG, clone 53A2), Anti phospho Rictor (pRictor; IgG, Thr1135, D30A3), Anti Raptor (IgG, clone 24C12), Anti phospho Raptor (pRaptor; IgG, Ser792), Anti p70s6k (IgG, clone 49D7) and Anti phospho p70s6k (pp70s6k; IgG, Thr389, clone 108D2) (all were obtained from New England Biolabs, Frankfurt, Germany), as well as Anti Akt (IgG1, clone 55) and Anti phospho Akt (pAkt; IgG1, Ser472/Ser473, clone 104A282) (both were obtained from BD Pharmingen).

To investigate histone acetylation, cell lysates were marked with anti-histone H3 (IgG, clone Y173), anti-acetylated H3 (IgG, clone Y28, dilution 1:500), anti-histone H4 (polyclonal IgG) and anti-acetylated H4 (Lys8, polyclonal IgG, dilution 1:500) (all were obtained from Biomol GmbH, Hamburg, Germany).

Gimp 2.8 software (https://www.gimp.org/) was used to perform pixel density analysis of the protein bands and to calculate the ratio of protein intensity/β-actin intensity.

### 2.7. siRNA Knockdown

PC3^par^ or PC3^res^ cells (3 × 10^5^/6-well) were transferred with small interfering RNA (siRNA) directed against cdk1 (gene ID: 983, target sequence: *AAGGGGTTCCTAGTACTGCAA*), cyclin B (gene ID: 891, target sequence: *AATGTAGTCATGGTAAATCAA*), rictor (gene ID: 253260, target sequence: *TCCGATCATGGGCAGGTATTA*) or raptor (gene ID: 57521, target sequence: *TCGGACGTGGCCATGAAAGTA*; all were obtained from Qiagen, Hilden, Germany) at a ratio of 1:6. Non-treated cells and cells with 10 nM control siRNA (all stars negative control siRNA; Qiagen) served as the controls. Subsequently, tumor growth was analyzed by the MTT assay as indicated above.

### 2.8. Statistics

All experiments were performed 3–6 times. Statistical significance was determined with the Wilcoxon–Mann–Whitney *U* test. Pixel density data were analyzed using the Student’s *t*-test. Differences were considered statistically significant at a *p*-value < 0.05. IC_50_ values were calculated with GraphPad Prism Plus software (version 5.0c; GraphPad Software Inc., La Jolla, CA, USA). 

## 3. Results

### 3.1. Tumor Growth and Proliferation

**Figure 1:**
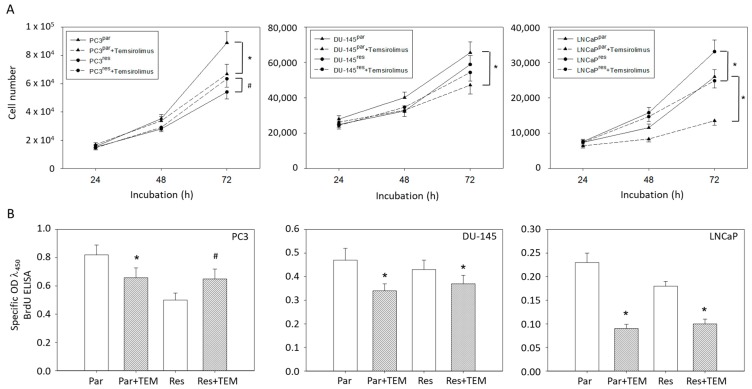
(**A**) Cell growth and (**B**) proliferation of PC3^par^/PC3^res^, DU-145^par^/DU-145^res^ and LNCaP^par^/LNCaP^res^ with and without (re)incubation with low-dosed (10 nM) temsirolimus. Measurements were done in triplicate and repeated five times. One representative experiment is shown. OD = optical density. * indicates significant down-regulation following temsirolimus (re)incubation, ^#^ indicates significant up-regulation following temsirolimus (re)incubation.

Based on MTT assays, more PC3^par^ cells were counted after 72 h incubation than PC3^res^ cells. However, the number of PC3^par^ cells significantly decreased in the presence of temsirolimus, whereas the number of PC3^res^ cells was elevated following low-dosed temsirolimus re-exposure (Figure 1A). The number of DU-145^par^ cells was above that of DU-145^res^ cells. Temsirolimus diminished growth of DU-145^par^ but not of DU-145^res^ cells. In contrast to PC3 and DU-145 cells, the number of drug-resistant LNCaP cells was higher than that of the respective sensitive subline. However, temsirolimus (re)treatment caused cell growth reduction of both LNCaP^par^ and LNCaP^res^ cells (Figure 1A).

The MTT data correlated with the BrdU incorporation test in as much as temsirolimus down-regulated proliferation of PC3^par^ but up-regulated proliferation of PC3^res^ cells (Figure 1B). Proliferation of DU-145^par^, LNCaP^par^ and (to a lesser extent) of DU-145^res^ and LNCaP^res^ was diminished by temsirolimus.

**Figure 2:**
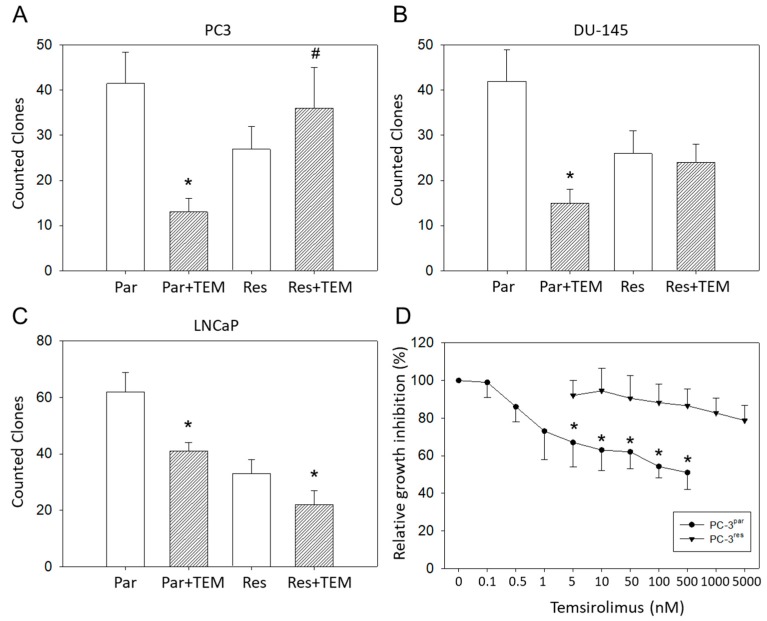
Clonal growth of (**A**) PC3^par^ versus PC3^res^, (**B**) DU145^par^ versus DU145^res^, and (**C**) LNCaP^par^ versus LNCaP^res^ with and without exposure to low-dosed (10 nM) temsirolimus (Par + TEM, Res + TEM). Experiments were done in triplicate and repeated five times. * indicates significant down-regulation following temsirolimus (re)incubation, ^#^ indicates significant up-regulation following temsirolimus (re)incubation. (**D**) Dose response of PC3^par^ and PC3^res^ cells to 72 h temsirolimus incubation (relative growth inhibition). Controls remained untreated and were set at 100%. * indicates significant difference.

Clonogenic growth data demonstrated a significant reduction in PC3^par^ clones but a considerable increase in PC3^res^ clones in the presence of temsirolimus, compared to untreated controls (Figure 2A). LNCaP^par^ and LNCaP^res^ clones became diminished as well as DU-145^par^ cell aggregates, but DU-145^res^ did not respond to temsirolimus re-treatment at all (Figure 2B,C).

Since PC3^res^ cells possessed the strongest resistance of the three evaluated cell lines, this cell line, along with its sensitive counterpart, PC3^par^, was used for further experiments. The IC_50_ values were 50.46 ± 11.01 nM temsirolimus for PC3^res^ versus 0.92 ± 0.18 nM temsirolimus for PC3^par^ (Figure 2D). The temsirolimus concentrations applied did not induce toxic effects as demonstrated by the trypan blue exclusion test. Apoptosis of both PC3^par^ and PC3^res^ cells was not significantly altered following temsirolimus application, as compared to the untreated controls.

### 3.2. Cell Cycle Analysis and Cell Cycle-Regulating Protein Expression

**Figure 3:**
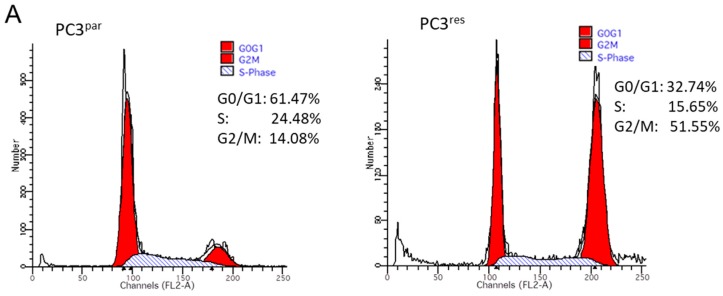

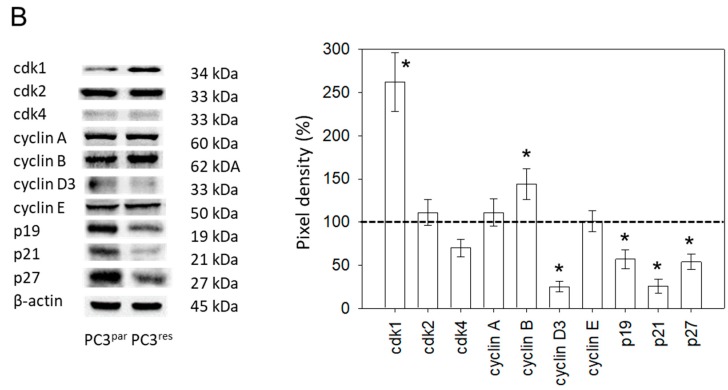
(**A**) Cell cycle and cell cycle-regulating proteins in PC3^par^ and PC3^res^ cells. Each cell population is expressed as percentage of total. One representative experiment of three is shown. (**B**) Western blot analysis of cell cycle proteins of PC3^par^ and PC3^res^. β-actin served as internal control. * indicates significant percentage difference between PC3^par^ and PC3^res^.

Resistance induction of PC3 cells was characterized by a strong distribution shift from the G0/G1 into the G2/M-phase (Figure 3A). This process was accompanied by distinct modifications of the expression profile of cell cycle-regulating proteins. Particularly, cyclin B, along with its binding partner cdk1, was elevated in PC3^res^, compared to PC3^par^ cells (Figure 3B). Cyclin D3 and the tumor suppressor proteins p19, p21 and p27 were all reduced in the resistant tumor cells (Figure 3B).

### 3.3. VPA Suppresses Growth of PC3^par^ and PC3^res^ Cells

**Figure 4:**
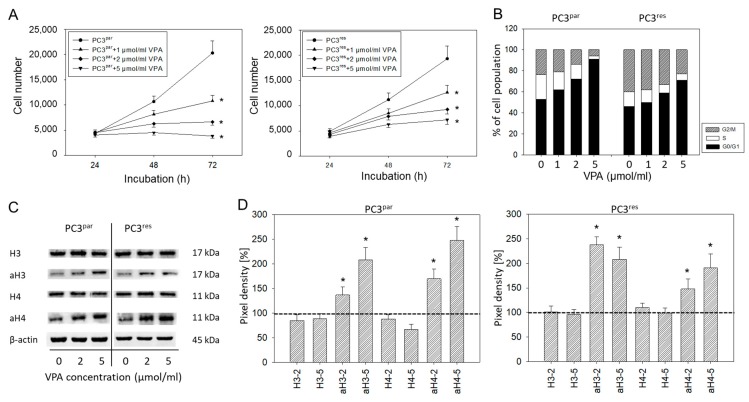
(**A**) Growth of PC3^par^ and PC3^res^ cells exposed to valproic acid (VPA). Controls remained untreated. Bars indicate standard deviation (SD). * indicates significant difference to untreated control cells. One representative of *n* = 6. (**B**) Cell cycle analysis of sensitive and resistant PC3 cells treated with VPA. Controls (0) remained untreated. One representative experiment of three. (**C**) Influence of VPA on histone expression level. β-actin served as the internal control. (**D**) Histone pixel density analysis. 2 = 2 µmol/mL VPA, 5 = 5 µmol/mL VPA. * indicates significant difference to untreated control cells.

Cell numbers of both PC3^par^ and PC3^res^ cells were significantly reduced by VPA, compared to their respective controls (Figure 4A). Cell cycle analysis revealed an increase in the tumor cells in G0/G1 following VPA exposure (Figure 4B). The effects were paralleled by up-regulated histone H3 and H4 expression levels (Figure 4C,D).

### 3.4. Influence of VPA on Cell Signaling Processes

**Figure 5:**
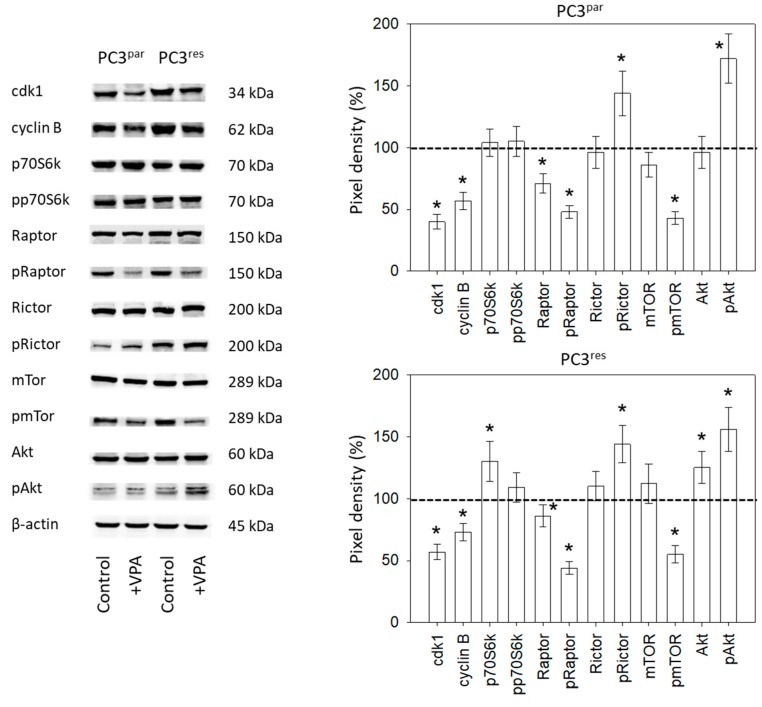
Protein expression profile of cell cycle-regulating and targeted proteins in PC3^par^ and PC3^res^ cells after three days exposure to VPA (1 µmol/mL) and untreated controls. β-actin served as the internal control. * indicates significant difference to untreated control cells.

Ongoing experiments concentrated on the cdk1-cyclin B axis, which was profoundly modified in the temsirolimus-resistant cell cultures, and on the Akt-mTOR signaling pathway, since this is the primary target of temsirolimus. The protein pmTOR with its sub-structures pRictor and pRaptor was strongly elevated in PC3^res^ cells, compared to PC3^par^. The upstream protein Akt was distinctly increased, whereas expression of pp70S6k was only slightly enhanced in PC3^res^ cells, compared to sensitive cells (Figure 5). Adding VPA to the cell cultures induced a loss of cdk1 and cyclin B in both sensitive and resistant tumor cells. Furthermore, pRaptor and pmTOR were suppressed in PC3^par^ and PC3^res^ cells. pRictor and pAkt were enhanced by VPA in both PC3^par^ and PC3^res^ cells.

### 3.5. Protein Knockdown Studies

**Figure 6:**
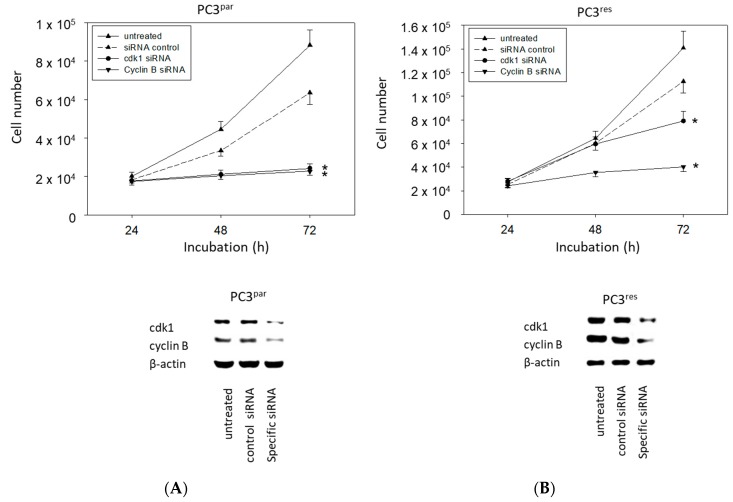
Cell growth after functional blocking with small interfering RNA (siRNA) targeting cdk1 and cyclin B of (**A**) PC3^par^ and (**B**) PC3^res^ cells. Controls remained untreated. Lower panel: Protein expression profile of cdk1 and cyclin B after functional blocking with siRNA. β-actin served as internal control. One representative of three separate experiments is shown. * indicates significant difference to control.

**Figure 7:**
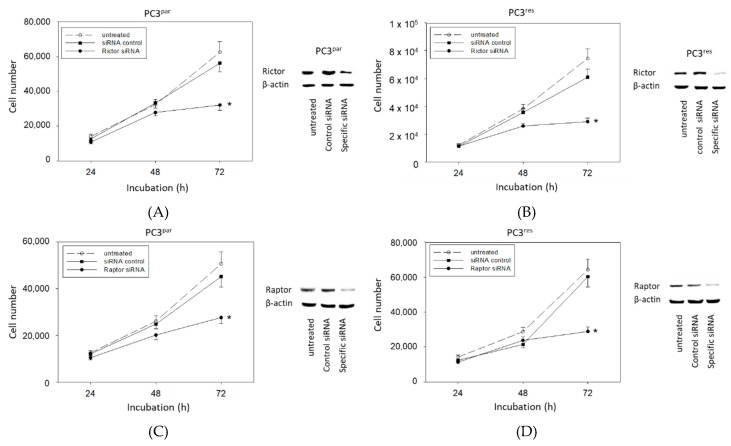
Functional blocking with siRNA targeting (**A**,**B**) Rictor and (**C**,**D**) Raptor of (**A**,**C**) PC3^par^ and (**B**,**D**) PC3^res^ cells. Controls remained untreated. Transfection efficacy is shown by Western blotting. β-actin served as internal control. One representative of three separate experiments is shown. * indicates significant difference to control.

The physiologic relevance of the intracellular proteins modified by VPA was further explored by siRNA knockdown studies. Successful knockdown was verified by Western blotting (Figure 6: cdk1, cyclin B; Figure 7: Rictor, Raptor). Both cdk1 and cyclin B suppression was associated with diminished cell growth of PC3^par^ and PC3^res^ cells (Figure 6). Knockdown of Rictor and Raptor also induced a significant cell growth reduction of both the drug-resistant and drug-sensitive PC3 cells (Figure 7).

## 4. Discussion

Of the three evaluated cell lines exposed to temsirolimus over 12 months, PC3 exerted resistance characteristics most strongly. This was evidenced by an elevated number of tumor cells in the G2/M-phase, associated with increased proliferative activity and colony formation, compared to its drug-sensitive counterpart. Everolimus-resistant PC3 cells have also shown increased mitosis. However, re-treatment of these resistant tumor cells with low-dosed everolimus did not further enhance cell proliferation, as occurred after temsirolimus re-treatment [16]. This difference may be clinically important, since temsirolimus seems to evoke resistance either more rapidly or more strongly than everolimus. However, this remains speculative, since a direct comparison has not yet been carried out.

Protein analysis pointed to a significant up-regulation of cdk1 and cyclin B in PC3^res^ versus PC3^par^ cells. The Cancer Genome Atlas (TCGA) database shows that elevated expression levels of cdk1 are associated with shorter disease-free survival of prostate cancer patients, and particularly of CRPC patients [17]. Accordingly, next-generation sequencing of CRPC signatures has revealed that cdk1 significantly predicts survival of patients with prostate cancer [18]. Based on a study involving metastatic and non-metastatic prostate cancer, cyclin B has been shown to reflect the highest sensitivity regarding metastasis prediction and clinical outcome [19]. Therefore, over-expression of the cdk1-cyclin B axis seems to contribute to the aggressive tumor regrowth caused by temsirolimus resistance and, consequently, this axis may provide a pivotal therapeutic target in counteracting resistance.

The cell cycle-related proteins p19, p21 and p27 were all found to be diminished in PC3^res^ cells, indicating that loss of these proteins may correlate with resistance induction. p19, p21 and p27 loss have also been detected in prostate cancer cells with acquired resistance towards the mTOR inhibitor everolimus [16]. Reduced p27 expression has also been found in temsirolimus-resistant bladder and renal cell carcinoma cell lines [13,14], as well as in breast cancer cells insensitive to both rapamycin and temsirolimus [20].

Since p21 and p27 have been reported to prevent the cyclin B-cdk1-driven progression of prostate cancer cells into G2/M [21], it is possible that long-term application of temsirolimus creates a negative feedback loop, causing suppression of p21 and p27, enhanced expression of cyclin B and cdk1, and accelerated mitotic progression.

VPA significantly hindered the growth of both PC3^par^ and PC3^res^ cells. Evaluation of the histone acetylation status revealed increased aH3 and aH4 expression in the resistant and sensitive cells, pointing to an epigenetic mechanism. It is remarkable that VPA acted on the resistant and sensitive cells equally well. Ruscetti et al. recently discovered that epithelial-mesenchymal plasticity is epigenetically regulated and that HDAC inhibition not only prevents metastatic CRPC development but also sensitizes castration-resistant mesenchymal-like tumor cells to androgen deprivation therapy [22]. Notably, selective deacetylation of histone H3 has been linked to oncogenic transformation, anchorage-independent growth, and escape from contact inhibition. Patients with deacetylated H3 have been associated with an aggressive tumor phenotype and poor prognosis [23].

Increasing histone H3 acetylation has been shown to allow the expression of a subset of androgen-induced target genes, including the cell cycle regulators p21 and p27 [24]. There is also evidence that elevating histone H3 acetylation may sensitize cancer cells to chemotherapeutics [25,26]. VPA promotes cisplatin sensitivity [27] and restores erlotinib [28], sorafenib [29], gemcitabine, and vincristine responsiveness [30,31]. Recent investigations demonstrate that VPA may also act on temsirolimus-resistant bladder cancer [13] and everolimus-resistant renal cell carcinoma cells [14]. Therefore, targeting epigenetic machinery may resensitize cancer cells to mTOR inhibitors. Indeed, VPA exposure strongly diminished mTOR and Raptor phosphorylation in PC3^res^ cells. Velásquez et al. have reported that cdk1 substitutes for mTOR [32], with the mTOR complex 1 (mTORC1) member, Raptor, being particularly involved in cdk1/cyclin B regulation [33]. It can therefore be expected that mTOR/Raptor suppression in VPA-treated prostate cancer cells is accompanied by a profound loss of cdk1 and cyclin B, as well. Since knocking down cdk1, cyclin B, or Raptor led to a significant reduction of tumor growth in the present study, it seems likely that diminishing these proteins may constitute a crucial mechanism (among others) explaining how VPA slows down the proliferative activity of prostate cancer cells.

Unexpectedly, pRictor as the pivotal component of mTORC2 and pAkt was up-regulated by VPA in both the drug-resistant and drug-sensitive cell lines. This finding is highly important. A murine prostate cancer model has shown epigenetic control of Rictor elevation that elicits unrestrained Akt signaling [34]. Rictor not only plays a crucial role in the development of prostate cancer [35] but also in promoting metastatic progression [34,36].

Experimental studies dealing with VPA–Rictor/Akt interaction in prostate cancer are sparse. Xia and coworkers recently reported that VPA suppresses Akt phosphorylation in PC3 cells, which is not concordant with our observation. However, in contrast to our investigation, VPA was applied by Xia at much higher concentrations of 2.5 and 5 mmol/L [37]. Based on the DU-145 prostate cancer cell line, evidence has been presented that HDAC–mTOR cross-linking exists in the tumor cells, whereby elevation of aH3 and aH4 is coupled to reduced pmTOR and pRaptor, but also to an enhanced pRictor and pAkt expression level [11]. Accordingly, a microarray analysis in an animal model has shown that VPA exposure decreased the mTOR but increased the Akt gene [38]. VPA also promoted an increase in the Akt phosphorylation level in mice [39].

The relevance of this cross-linking is not yet clear. Although VPA potently counteracted temsirolimus resistance by blocking growth and proliferation of PC3^res^ cells, it is not possible to rule out a negative feedback loop being created, lowering the efficacy of VPA. Although this is speculative, chronic treatment of RCC cells with VPA has been shown to cause a significant enhancement of Akt and mice xenotransplanted with RCC cells, not responding to VPA, have been characterized by a high Akt expression level [40].

A large population-based study has strongly implicated a lowered risk of prostate cancer among users of VPA [41], and recent clinical trials of the HDAC inhibitors vorinostat, romidepsin, and panobinostat have provided cautious optimism towards improved outcomes for CRPC patients [42]. Nevertheless, HDAC inhibitors administered as single agents have failed to show convincing activity in combating CRPC [43,44]. With reference to the present findings, this could be partially explained by the mode of action of HDAC inhibitors, revealing an activation of Rictor and Akt. This mechanism has only been demonstrated for VPA. It must be clarified whether such activation is restricted to this particular HDAC inhibitor or is transferrable to other HDAC inhibitors as well.

Since VPA administration reversed acquired resistance towards temsirolimus in prostate cancer cells, combined application of an mTOR and HDAC inhibitor may be a novel concept in treating CRPC patients. However, due to the undesired modification of Akt and Rictor by VPA, an additional drug targeting Akt should possibly be added to the treatment regimen. Simultaneous application of the dual PI3K/mTOR inhibitor BEZ235 and the HDAC inhibitor trichostatin A (TSA) synergistically suppressed growth and metastasis of lung cancer cells in vitro and in vivo by inactivating both Akt and mTOR and down-regulating HDAC [45]. No effect or even an enhancement of pAkt in breast cancer cells has been induced by 50 nM TSA. However a strong decline of pAkt, along with a considerable reduction of cell viability, has been seen when TSA was combined with an HDAC inhibitor blocking complexes of mTOR, mTORC1 and mTORC2 [46].

VPA administration has been shown to counteract temsirolimus resistance in prostate cancer cells by reducing cdk1/cyclin B and the mTORC1 member Raptor. Elevation of Akt and Rictor by VPA may point to an undesired feedback mechanism, which must be considered in the clinical setting. A combination therapy, consisting of an mTOR inhibitor, an HDAC inhibitor and a selective Akt inhibitor, may be an innovative concept to fight CRPC. Ongoing animal studies are the next step to validate the in vitro data presented here.

## 5. Conclusions

Based on a prostate cancer cell model, evidence is presented here indicating that epigenetic modulation of the tumor cells by an HDAC-inhibitor could provide a novel strategy to combat progressive prostate cancer. This might be particularly true for treating tumor patients with acquired drug resistance. Whether combined targeting HDAC, Akt and mTOR may prevent or delay resistance development is not clear yet. Therefore, this treatment protocol should be evaluated in future studies.
